# Deep learning prediction of stroke thrombus red blood cell content from multiparametric MRI

**DOI:** 10.1177/15910199221140962

**Published:** 2022-11-28

**Authors:** Spencer D Christiansen, Junmin Liu, Maria Bres Bullrich, Manas Sharma, Melfort Boulton, Sachin K Pandey, Luciano A Sposato, Maria Drangova

**Affiliations:** 1 Robarts Research Institute, Western University, London, Ontario, Canada; 2 Department of Medical Biophysics, Schulich School of Medicine & Dentistry, 6221 Western University, London, Ontario, Canada; 3 Department of Clinical Neurological Sciences, 6221 Western University, London, Ontario, Canada; 4 Department of Medical Imaging, 6221 Western University, London, Ontario, Canada

**Keywords:** Ischemic stroke, MRI, thrombus imaging, RBC, deep learning, machine learning

## Abstract

**Background and purpose:**

Thrombus red blood cell (RBC) content has been shown to be a significant factor influencing the efficacy of acute ischemic stroke treatment. In this study, our objective was to evaluate the ability of convolutional neural networks (CNNs) to predict ischemic stroke thrombus RBC content using multiparametric MR images.

**Materials and methods:**

Retrieved stroke thrombi were scanned *ex vivo* using a three-dimensional multi-echo gradient echo sequence and histologically analyzed. 188 thrombus R_2_*, quantitative susceptibility mapping and late-echo GRE magnitude image slices were used to train and test a 3-layer CNN through cross-validation. Data augmentation techniques involving input equalization and random image transformation were employed to improve network performance. The network was assessed for its ability to quantitatively predict RBC content and to classify thrombi into RBC-rich and RBC-poor groups.

**Results:**

The CNN predicted thrombus RBC content with an accuracy of 62% (95% CI 48–76%) when trained on the original dataset and improved to 72% (95% CI 60–84%) on the augmented dataset. The network classified thrombi as RBC-rich or poor with an accuracy of 71% (95% CI 58–84%) and an area under the curve of 0.72 (95% CI 0.57–0.87) when trained on the original dataset and improved to 80% (95% CI 69–91%) and 0.84 (95% CI 0.73–0.95), respectively, on the augmented dataset.

**Conclusions:**

The CNN was able to accurately predict thrombus RBC content using multiparametric MR images, and could provide a means to guide treatment strategy in acute ischemic stroke.

## Introduction

Acute ischemic stroke (AIS) treatment aims to achieve rapid recanalization, yet despite improvements in thrombolytic agents and reperfusion devices successful recanalization is not achieved in approximately 25% of patients.^1^ Thrombus composition has emerged as a significant factor influencing the ultimate success of AIS treatment.^2^ Particularly, the proportion of thrombus red blood cell (RBC) content has been linked to the efficacy of both tPA and endovascular thrombectomy (EVT) therapies.^3,4^ Non-invasive prediction of AIS thrombus RBC content using medical imaging would provide a valuable tool by enabling personalization of AIS treatment by stroke interventionalists.

The presence of qualitative imaging signs, such as the susceptibility vessel sign in MRI and hyperdense artery sign in CT, have established the ability of imaging to characterize RBC content within thrombi categorically, distinguishing between groups such as RBC vs. fibrin-dominant.^4,5^ However, such signs have proven inconsistent in their predictions of thrombus amenability to tPA and EVT therapies,^6^ and have noted issues with inter-scanner variability.^7^ Quantitative imaging techniques utilizing MR R_2_* and quantitative susceptibility mapping (QSM) values, or CT Hounsfield units have shown promise for robust, quantitative prediction of RBC content *in vitro*,^8–10^ and have been associated with efficacy of tPA and EVT therapies.^11,12^ However, these techniques remain limited by their use of a single imaging value or metric to represent entire thrombi. Histological studies have consistently demonstrated that thrombi are heterogeneous and complex in structure,^13^ and that complexities such as the distribution of fibrin and RBC components have an impact on treatment efficacy.^14^

To account for these structural complexities, machine learning techniques have recently started to be applied to thrombus analysis. Models generated from MR or CT imaging texture features have demonstrated sensitivity to clot RBC content *in vitro*^15,16^ and an ability to predict clinical features such as response to tPA.^17^ Concerns linger however regarding the reproducibility of calculated texture features and the generalizability of these techniques.^18^ Furthermore, machine learning techniques are limited by the need to initially handpick extractable features for study; the histomorphological basis underlying the distinguishing ability of imaging texture features remains an area of ongoing research.^19^

Deep learning neural networks provide a complimentary tool to machine learning techniques with the primary advantage being that featurization, the process of turning raw signal into a modellable predictor, is fully automated. Convolutional neural networks (CNNs) have been successfully applied towards medical imaging problems where relevant imaging features are difficult to define, such as for predicting brain age and assessing aneurysm stability.^20,21^ CNNs have also demonstrated utility related to AIS, in areas such as predicting ischemic tissue fate and thrombus detection.^22,23^ However, CNNs have not yet been applied towards analysis of the thrombus itself. In this study, we apply a CNN to predict AIS thrombus RBC content from multiparametric MR images. Specifically, we trained a CNN on thrombus MR R_2_*, QSM and T_2_*-weighted gradient echo (GRE) images acquired *ex vivo* following retrieval by EVT, investigated the use of data augmentation to improve network performance, and assessed the accuracy of the trained CNN for quantitative and qualitative prediction of thrombus histological RBC content.

## Materials and methods

### Thrombus collection and storage

Institutional research ethics board approval was obtained for this study. AIS patients treated with EVT at the local stroke centre were consecutively enrolled between the periods of February 2016 to November 2017. Patients were excluded if insufficient thrombus material was retrieved or if younger than 18 years old. Informed consent was obtained following the EVT procedure and samples were discarded if consent was refused.

A total of 109 thrombi were collected from 65 AIS patients. Prior to *ex vivo* MR imaging, retrieved thrombi were stored inside a 150 mL plastic jar containing a non-adherent pad (Telfa; Covidien, Mansfield, MA) wetted with heparinized saline to retain moisture. Those retrieved during working hours were kept at room temperature and scanned within 6 h of retrieval, otherwise thrombi were kept inside a refrigerator overnight until the following workday. It was observed that thrombi which had been placed in a fridge and stored overnight had significantly lower R_2_* and QSM values than those kept at room temperature (Supplementary Figure 1). Thus, only those stored at room temperature were included in the study; final sample size was 48 thrombi. Clinical details associated with each group of stored thrombi are listed in Table 1.

**Table 1.** Clinical details of the patient cohort for each thrombus storage group.

Storage type	Room temperature (n = 31)	Refrigerator (n = 32)
Age (mean ± SD)	67 ± 17	71 ± 12
Sex, female	14 (45%)	14 (44%)
Number of thrombi	48	61
Etiology		
Large artery atherosclerosis	6 (20%)	3 (9%)
Cardioembolism	21 (68%)	22 (69%)
Dissection	2 (6%)	1 (3%)
Undetermined	2 (6%)	6 (19%)
Occlusion site		
MCA	25 (81%)	27 (85%)
ICA	1 (3%)	3 (9%)
Vertebrobasilar	5 (16%)	2 (6%)
EVT technique		
Stent	26 (84%)	29 (91%)
Aspiration	5 (16%)	3 (9%)
IV tPA	15 (48%)	11 (34%)

MCA: middle cerebral artery; ICA: internal carotid artery; EVT: endovascular thrombectomy; IV tPA: intravenous tissue plasminogen activator

### Ex vivo thrombus imaging

Thrombi were scanned inside 1 cm diameter polystyrene vials containing porcine plasma and vertically inserted into a 15-cm diameter agar-filled container.^8^ When more than one specimen was obtained from a single participant due to fragmentation, thrombus pieces were scanned in individual tubes and analyzed separately. Scanning was performed at 3.0 T using a whole-body MRI scanner (GE 750; GE Medical Systems, Milwaukee, WI) with a 32-channel receive head coil. Scans were acquired using a 3D multi-echo bipolar GRE sequence designed for rapid clinical imaging *in vivo*^24^; the sequence includes two 5-echo trains: the first train was optimized for chemical shift imaging (first TE = 3.20 ms, echo spacing = 1.46 ms); the second train was optimized to highlight susceptibility-related contrast (first TE = 16.75 ms, echo spacing = 7.15 ms). The remaining scan parameters were TR = 47.6 ms, bandwidth = 142.9 kHz, flip angle = 10°; field of view = 18 cm; matrix size = 192 × 192 × 36, for a final voxel dimension of 0.94 × 0.94 × 1.0 mm^3^. Total acquisition time was 5 min 33 s. Balanced steady-state gradient echo (FIESTA-C) images with identical resolution and bandwidth were also acquired to facilitate thrombus segmentation (TE^­^ = 3 ms, TR = 6.1 ms, flip angle: 40°, phase cycles = 4, scan time = 2 min 47 s). All scans were acquired in the coronal plane, with the phantom laying flat along the MR table and vials aligned perpendicular to B_0_.

### Image processing

Image reconstruction was performed in Matlab (Matlab R2019a; Mathworks, Natick, MA) using the Orchestra Software Development Kit (GE Healthcare; Milwaukee, WI). Complex channel combination was performed using singular value decomposition from which GRE magnitude images were derived. R_2_* and QSM maps were generated from the complex channel combined data using the B0-NICE and MEDI QSM algorithms, respectively.^25,26^ Thrombi were segmented from the FIESTA-C images, which were inherently co-registered with the GRE sequence, using an in-house semiautomated Matlab code. Thrombus R_2_*, QSM and GRE magnitude pixel values were each separately z-score normalized according to distributions derived from all segmented thrombi.

### Histological analysis

Immediately following imaging, thrombi were fixed in 10% formalin, embedded in paraffin and arranged for sectioning along the MR slice-encoding direction. Thrombi were sectioned at 5 μm thickness and stained with hematoxylin & eosin. The first 14 were sectioned in 4 evenly spaced regions throughout each thrombus; it was observed that RBC content varied minimally between thrombus slices, concurring with the findings of Staessens *et al*.,^27^ so the remaining thrombi were sectioned only once through the middle of each sample (Supplementary Figure 2). Stained slides were scanned at × 40 magnification. The color segmentation plugin (EPFL, Lausanne, Switzerland) in ImageJ (National Institutes of Health, Bethesda, MD) with post-processing correction for outlying pixels in Matlab was used to quantify thrombus RBC percentage by area.

Thrombi were categorized into RBC-rich or RBC-poor groups based on whether their quantified RBC content was above or below the median percentage obtained from all thrombi.

### Deep learning network

#### Network architecture

Using the acquired thrombus MR images, a CNN was trained in Matlab to predict histological RBC content. Two-dimensional slices of the segmented, normalized R_2_*, QSM and late echo GRE magnitude images (TE = 31 ms) were fed into the network as 3-channel images; each image was 49 × 56 × 3 pixels in size. 188 segmented 3-channel MR slices were available from the 48 analyzed thrombi for training and testing. It was assumed that all MR slices from a single thrombus had the same RBC content derived from histology. The network contained 3 convolutional layers and its architecture is depicted in Supplementary Figure 3. The number of feature channels for the convolutional layers was set to 16, 16, and 32, respectively. Network parameter weights were trained through minimization of the half mean squared error using the Adam optimization algorithm.

#### Data augmentation

Given the relatively small thrombus MR slice dataset available for network evaluation, overfitting of the training data was a concern. In light of this, image data augmentation was implemented in order to observe its effect on network performance. Augmentation consisted of three distinct components: input sampling equalization, image transformation, and dataset duplication.

**Figure 1.**
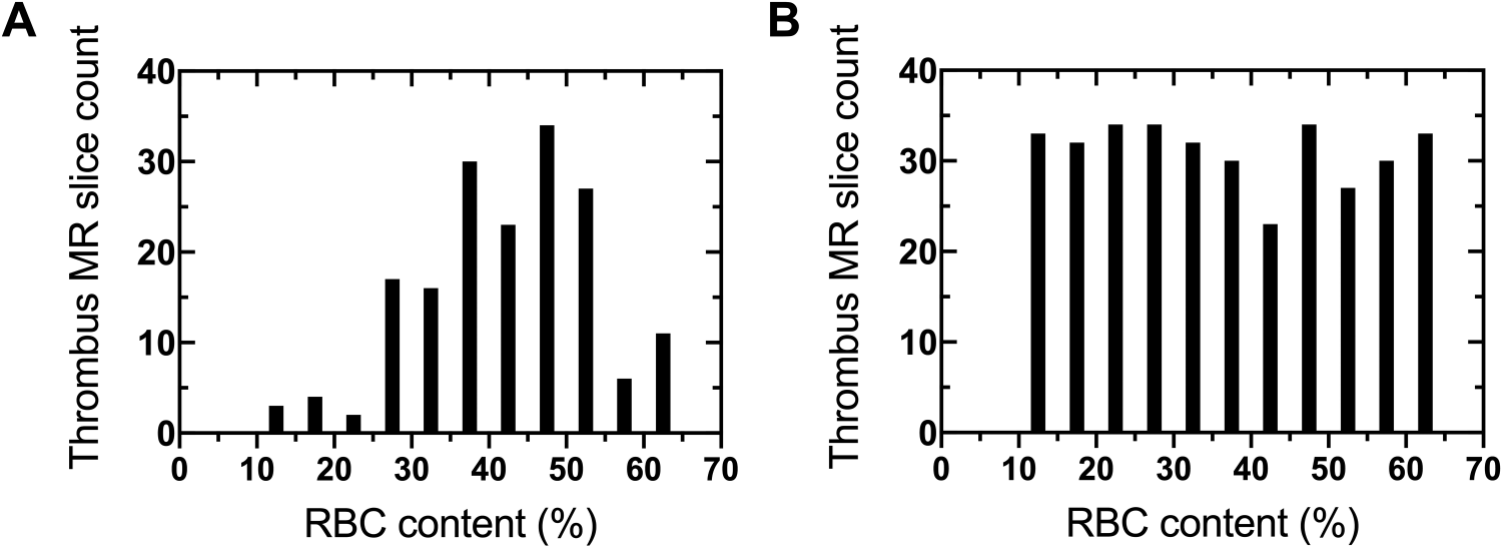
Representative training set thrombus RBC content distribution (A) before and (B) after input sampling equalization.

For input sampling equalization, oversampling of training data was performed to equalize the distribution of thrombus RBC content. Thrombus MR slices randomly selected to be in the training set were first binned based on RBC content in 5% intervals, and underrepresented bins had slices repeatedly sampled until their count equaled the nearest integer multiple less than or equal to that of the most represented bin. This resampling strategy is similar to the class-aware sampling strategy that has previously been employed on categorical data.^28^ A representative training set RBC distribution before and after input sampling equalization is shown in Figure 1.

**Figure 2.**
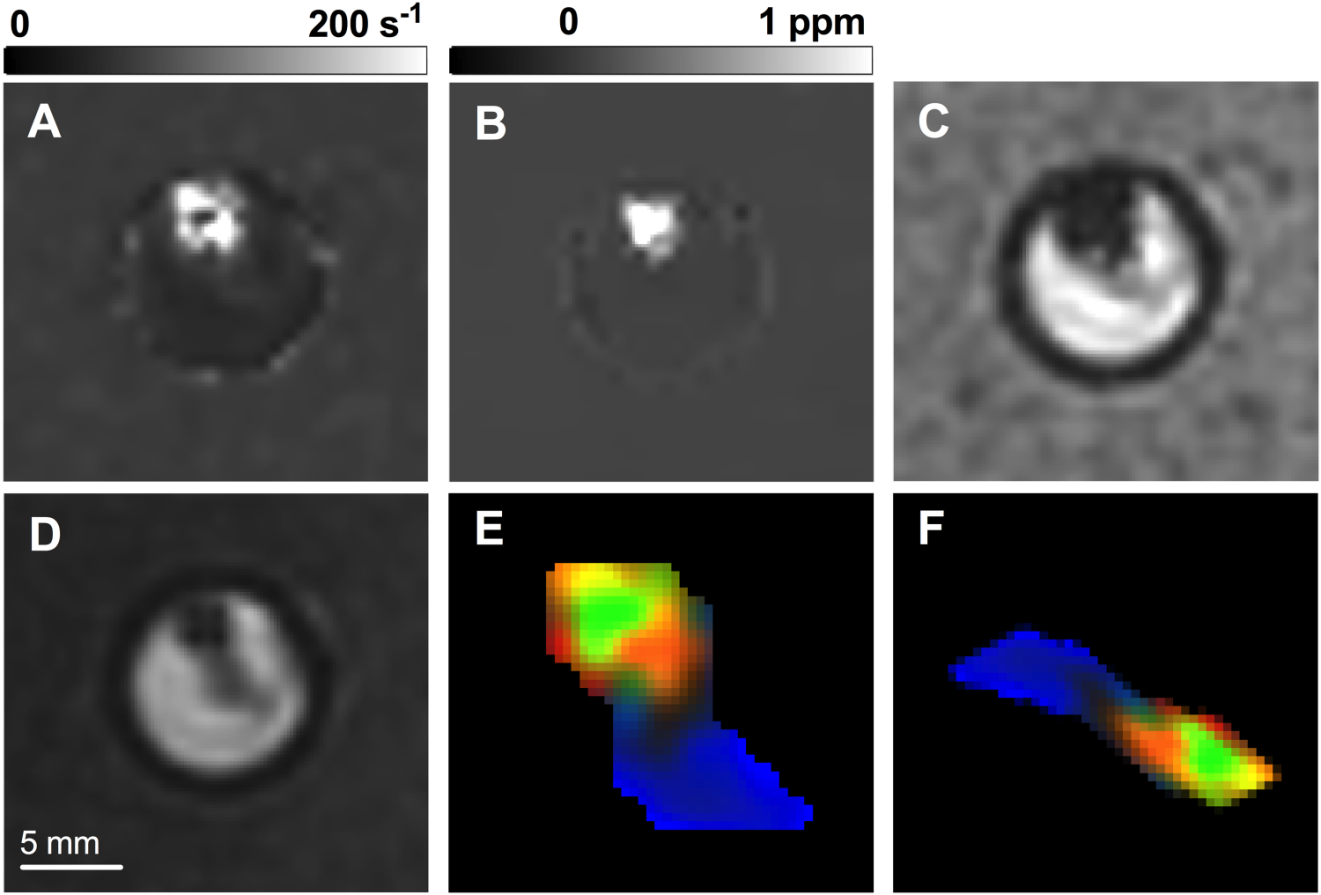
An example thrombus (A) R_2_*, (B) QSM, (C) GRE magnitude image slice, shown inside its scanning vial, along with the (D) FIESTA-C image used for segmentation. The resulting normalized, segmented thrombus 3-channel image slice used as CNN input is shown (E) before and (F) after random image transformation. R_2_*, QSM and GRE magnitude pixel values represent the red, green and blue image color channels, respectively.

For image transformation, a group of geometric transformations were randomly applied to the training dataset. Random transformations were performed within predefined ranges: rotations between −90 to 90°, integer vertical and horizontal translations up to 5 pixels, reflections across the X and Y axis, scaling between 0.7 to 1.3, and shearing between −30 to 50°. An example 3-channel thrombus input slice before and after transformation, along with its base MR images, is shown in Figure 2.

Finally, for dataset duplication the entire training set was copied by a × 5 multiple prior to network training. Duplication was implemented prior to image transformation so that all slices seen by the network were unique.

#### Network evaluation

The network was evaluated using random 8-fold cross validation, where the mean accuracy (proportion of predictions within 10% of histology) and absolute error for slices within the test set across all folds were used for network assessment. Overall thrombus RBC content predictions were derived from the median prediction of all available image slices, and were used to categorize thrombi into RBC-rich or RBC-poor groups to assess the ability of the networks to perform qualitative thrombus classification. Care was taken to ensure no slices associated with the same thrombus were mixed between the training and testing subsets within each cross-validation fold. Augmentation was performed only on images used for network training. The network was separately trained and evaluated using the original dataset and on the augmented dataset derived from the aforementioned augmentation scheme. In each case, the optimal network batch size, learning rate, number of training epochs, L2 regularization parameter (λ) and dropout rate were determined through a grid search optimized over network mean absolute error; optimal parameters are listed in Supplementary Table 1. The training time for a single 8-fold cross validation experiment was 86 s for the original dataset and 32 min for augmented dataset on a dual-core 2.8 GHz CPU (Intel Core i5) with 16 Gb of RAM.

#### Statistical analysis

In addition to accuracy and absolute error, network performance was also evaluated by the correlation between histological and predicted RBC content, using Pearson's r and slope, and by sensitivity, specificity and area under the receiver operating characteristic (ROC) curve (AUC) for distinguishing between RBC-rich and RBC-poor thrombi. Direct linear correlation between quantitative imaging values and RBC content was also performed to allow comparison with standard regression models. Statistical analysis was performed using GraphPad Prism (v8.2.1; GraphPad Software, La Jolla, CA).

## Results

The median thrombus RBC content determined from histological analysis was 38% (IQR: 30–49%; min: 12%, max: 61%). The median thrombus R_2_* value was 39 s^−1^ (IQR: 24–101 s^−1^; min: 16 s^−1^, max: 230 s^−1^), while the median QSM value was 0.017 ppm (IQR: −0.007–0.26 ppm; min: −0.12 ppm, max: 1.6 ppm). There was no correlation between either R_2_* (r = 0.14) or QSM (r = 0.09) values and thrombus RBC content (Supplementary Figure 4). The network trained on the original dataset was able to predict RBC content with an accuracy and absolute error of 62% (95% CI 48–76%) and 8.3% (95% CI 6.5–10.1%), respectively. For the network trained on the augmented dataset the accuracy and mean absolute error improved to 72% (95% CI 60–84%) and 8.1% (95% CI 6.3–9.9%), respectively. For reference, a naive prediction (prediction of the median overall histological RBC percentage for all thrombi) would produce an accuracy and absolute error of 48 and 10.4%, respectively. There was no correlation between thrombus size and network performance in either the original or augmented networks (Supplementary Figure 5).

**Figure 3.**
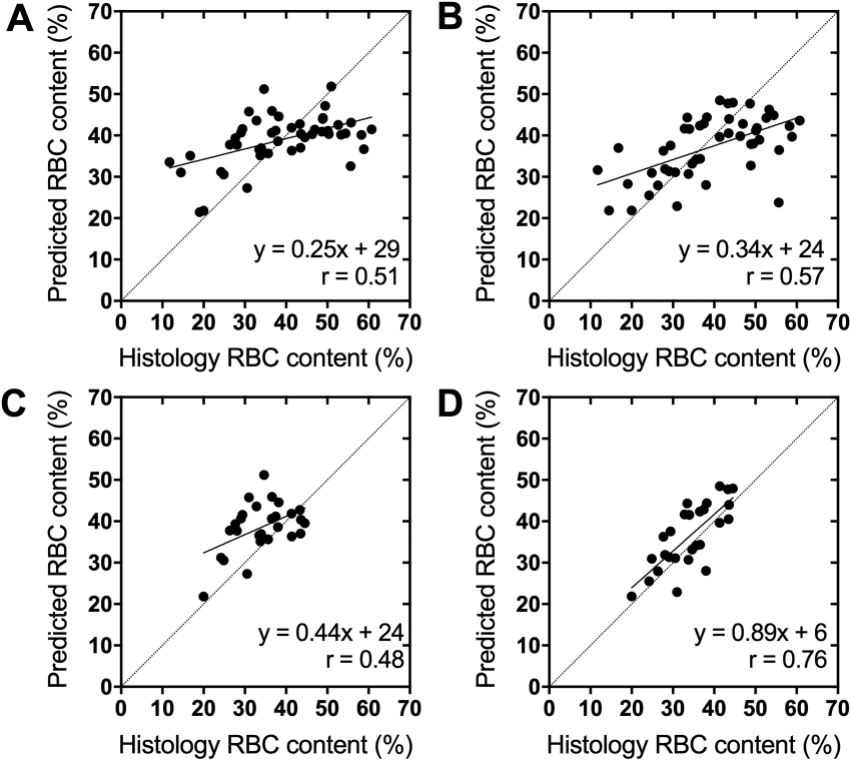
Linear regression plots of the CNN predicted thrombus RBC content against the histological value for the networks trained on the (A) original and (B) augmented datasets. Plotted are the median predictions from all MR slices available for each thrombus. These same predictions replotted with only thrombi with histological RBC content between 20–45% are shown in (C) and (D), respectively.

Regression curves plotting the predicted against histologically determined thrombus RBC content were used to determine correlation coefficients and linear regression slopes. Correlation coefficients and regression slopes derived from the original network's predictions improved from 0.51 (95% CI 0.27–0.69) to 0.57 (95% CI 0.34–0.73) and 0.25 (95% CI 0.13–0.37) to 0.34 (95% CI 0.19–0.48), respectively, in the augmented network. Regression plots from each network are shown in Figure 3(A) and (B). Despite only modest improvements in correlation and slope with data augmentation, the distribution of RBC content predictions in the augmented dataset is noticeably more linear than the largely flat distribution produced by the original dataset.

In general, each network predicted RBC content more accurately for thrombi with histological values closer to the median. When the thrombi included in the regression plots are limited to a narrower range of RBC content (20–45%), slopes of the prediction curves increase substantially, reaching up to 0.89 (95% CI 0.58–1.19) in the network trained on the augmented dataset (Figure 3(C) and (D). Performance of the networks trained on the original and augmented dataset on all thrombi and when limited to thrombi between 20–45% RBC content are shown in Table 2. The difference in network performance between the original and augmented datasets is more pronounced within this trimmed subset of predictions, with accuracy improving from 74% (95% CI 74 61–88) to 96% (95% CI 91–100) and absolute error improving from 6.3% (95% CI 4.5–8.0) to 4.7% (95% CI 3.5–5.8) after data augmentation. The increased performance within this subset compared to that achieved on the entire dataset suggests that both networks were hindered by the limited data available for thrombi with less common RBC content, and that the exceptional performance achieved on this subset may be broadly realizable if the network were trained on a larger dataset.

**Table 2.** Performance of the CNN for histological RBC content prediction on all thrombi and when limited only to thrombi between 20–45% RBC content (95% CI).

Training set	Accuracy (%)	Absolute error (%)	R	Slope
Original	62 (48–76)	8.3 (6.5–10.1)	0.51 (0.27–0.69)	0.25 (0.13–0.37)
Augmented	72 (60–84)	8.1 (6.3–9.9)	0.57 (0.34–0.73)	0.34 (0.19–0.48)
Original (20–45%)	74 (61–88)	6.3 (4.5–8.0)	0.48 (0.12–0.73)	0.44 (0.13–0.76)
Augmented (20–45%)	96 (91–100)	4.7 (3.5–5.8)	0.76 (0.52–0.88)	0.89 (0.58–1.19)

CNN: convolutional neural network; RBC: red blood cell.

**Figure 4.**
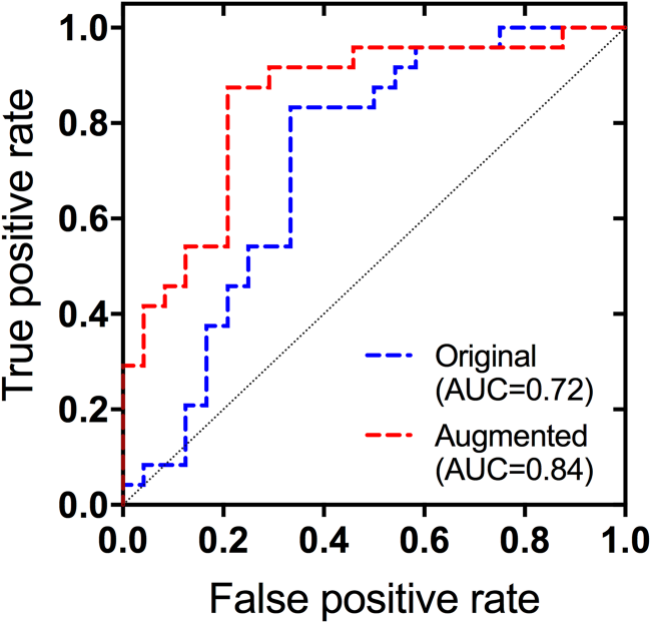
ROC curves for the identification of RBC-poor thrombi based on the quantitative RBC content predictions of the network trained on the original and augmented datasets.

Quantitative thrombus RBC content predictions were further used to directly categorize thrombi into RBC-rich and RBC-poor groups based on whether predictions were above or below the median RBC value (38%). The network trained on the original dataset correctly classified 71% (95% CI 58–84%) of thrombi as RBC-rich or poor, and improved to 80% (95% CI 69–91%) in the augmented network. ROC curves detailing each network's ability to differentiate between the thrombus groups are shown in Figure 4, while performance metrics are given in Table 3. The network identified RBC-poor thrombi with an AUC of 0.72 (95% CI 0.57–0.87) and improved to 0.84 (95% CI 0.73–0.95) when trained on the augmented dataset. There was no significant difference in mean imaging values between RBC-rich and poor clots in R_2_* (79 vs. 54 s^−1^; *p* = 0.14) or QSM (0.17 vs. 0.18 ppm; *p* = 0.9), however there was a trend towards higher median imaging values in the RBC-rich group compared to the RBC-poor and 20–45% RBC subsets (Supplementary Table 2).

**Table 3.** Performance of the CNN for identifying RBC-poor thrombi using quantitative predictions of RBC content (95% CI).

Training set	Accuracy (%)	AUC	Sensitivity (%)	Specificity (%)
Original	71 (58–84)	0.72 (0.57–0.87)	67 (48–86)	83 (68–98)
Augmented	80 (69–91)	0.84 (0.73–0.95)	79 (63–95)	88 (75–100)

AUC: area under the receiver operating characteristic curve; CNN: convolutional neural network; RBC: red blood cell.

## Discussion

In order to evaluate the ability of CNNs to predict AIS thrombus RBC content, we trained a 3-layer CNN on multiparametric thrombus MR images. Even with a modest dataset of 48 thrombi (188 slices) for training, the CNN was capable of predicting RBC content in AIS thrombi with a mean absolute error of approximately 8%. This was achieved by using data augmentation strategies designed to increase the effective size of the dataset and reduce overfitting. On the augmented dataset, the network was able to differentiate between RBC-rich and RBC-poor thrombi with an accuracy of 80%.

Our CNN was trained on *ex vivo* images of retrieved AIS thrombi, which were acquired using an imaging protocol similar to that of previous *in vitro* blood clot imaging experiments^8,9^ and *ex vivo* thrombus MR microscopy studies.^29^ Due to the limited size of our dataset, we investigated the use of data augmentation for improving CNN performance. Data augmentation is a widely used method to improve deep learning network performance, largely achieved through reducing overfitting of the training data.^30^ Here, we oversampled our dataset such that thrombi of all RBC contents were equally represented in the training dataset. Input sampling equalization through oversampling is one of the most common methods for dealing with class imbalance in deep learning, and forces the network to learn features relevant to the entire dataset rather than only the most common entries.^31^ Additionally, we applied a multitude of random image transformations to our dataset, which has shown to outperform single transformations applied on their own.^32,33^ We used geometric transformations specifically to avoid the network focusing on irrelevant characteristics of the thrombi such as orientation and shape, which can be altered during thrombectomy. Finally, we increased the size of the training dataset directly by duplicating the entire set prior to image transformation, which has been shown to improve the performance of CNNs trained on small datasets.^33^ The use of this larger, augmented dataset for network training resulted in an approximately 10% improvement in accuracy for both quantitative RBC content prediction and qualitative discrimination between RBC-rich and poor thrombi. The heightened performance of the network for thrombi with RBC content close to the median (between 20–45%) suggests that an improvement in network performance for all thrombi could be expected if trained with a larger dataset.

With the use of the augmented dataset for training, the CNN was able to quantitatively predict RBC content in thrombi with approximately 72% accuracy and 8% mean absolute error. It is difficult to precisely define the level of accuracy required for a thrombus RBC content predictor to be clinically useful. Studies examining the relationship between histological thrombus RBC content and successful recanalization following EVT have found an average RBC content difference of approximately 17%,^3,34^ while in one study the RBC content difference between tPA-resistant thrombi and those untreated was approximately 10%.^35^ The mean absolute error of 8% achieved in this study, being smaller than the difference between these groups, suggests that our model may be sufficiently accurate to allow prediction of response to EVT and tPA treatments. RBC content predictions could be included into an AIS treatment response prediction model that incorporates clinical variables,^36^ or our CNN could be retrained to predict treatment response directly. RBC content predictions can also be used to categorize thrombi into RBC-rich and RBC-poor groups. Thrombi divided into histological groupings based on RBC content have been shown to significantly differ in EVT and tPA responsiveness, with RBC-rich thrombi being more likely to recanalize, while RBC content may also influence the relative success of different EVT device types.^4,34,37^ A binary model built to indicate the likely thrombus RBC content group could be used as a straightforward metric for clinicians to inform treatment strategy.

In this study no significant difference was found in imaging values between RBC-rich and RBC-poor thrombi, nor was any correlation identified between imaging values and thrombus RBC content. A recent thrombus MR imaging study performed *in vivo* similarly found no significant difference in R_2_* values between RBC-rich and poor thrombi, however they did identify a weak correlation between R_2_* and RBC content (r = 0.41).^38^ Though thrombus R_2_* and QSM values are sensitive to RBC content, both values are strongly influenced by RBC oxygenation.^39^ In our study, identifying a trend in the median but not mean imaging values across thrombus subsets suggests the presence of outliers, possibly in the form of uncommonly oxygenated or deoxygenated thrombi. Thrombus oxygenation levels could be affected by storage prior to scanning, or may be biased from including only those retrievable by EVT. Nonetheless, our model was able to differentiate between RBC-rich and poor thrombi despite no direct correlation observed between imaging values and histology.

Though our network of course needs to be validated on *in vivo* MR images of stroke thrombi before its ability to inform stroke treatment is assessed, the results obtained here remain promising. A prior machine learning model built using texture features extracted from *in vivo* thrombus CT images identified RBC-poor thrombi with an AUC of 0.84,^40^ equivalent to that achieved by our network despite having a much larger sample size available for training (N = 112). Furthermore, there is reason to believe that our network performance may improve when trained on *in vivo* images, as alterations to thrombi during retrieval or storage that could impact imaging results, as occurred in approximately half of our initial dataset requiring exclusion, will no longer be present. The sequence used in this study has been previously applied to healthy subjects *in vivo*, and is able to provide whole head coverage with scan times of less than 5 min, critical for compatibility with imaging of AIS patients.^24^

This study has a number of limitations. We made the assumption that all MR imaging slices for a given thrombus had an RBC content equal to the single mean value derived from histology. While there is evidence to suggest that composition varies minimally throughout thrombi,^27^ slight variations in RBC content across each MR slice would limit the CNN's accuracy. We used dataset duplication and 8-fold cross validation to evaluate our network instead of defining a separate test set. Doing so means the generalizability of the network may be overestimated, but cross-validation has been recommended for datasets too small to include a test set of meaningful size.^41^ Finally, we performed our imaging *ex vivo* after thrombi had been retrieved through EVT. This allowed us to study human thrombi without impacting the course of patient treatment, but introduces confounding factors which could affect imaging results such as the effect of thrombus storage outside the body, potential alterations in thrombus structure during retrieval and tPA administration, and differences in temperature and field inhomogeneity relative to *in vivo* imaging. Performing the study *ex vivo* also meant that samples were limited to stroke thrombi which were retrievable by EVT and did not resolve following tPA administration. While evaluation of this technique *in vivo* with a larger sample size is ultimately required to demonstrate utility in AIS patients, the results presented here suggest that CNNs are capable of accurately predicting stroke thrombus RBC content and could enable imaging predictions of AIS thrombus treatment response to be performed.

## Conclusions

A 3-layer CNN is capable of RBC content prediction in AIS thrombi using R_2_*, QSM and late-echo GRE magnitude MR images. Improved CNN performance was achieved by employing data augmentation, and the trained network predicted thrombus RBC content with an absolute error of 8% and differentiated between thrombus RBC-rich and poor groups with an accuracy of 80%. This technique has the potential to enable prediction of thrombus RBC content in AIS patients, and ultimately allow thrombus composition to inform acute stroke treatment strategy.

## Supplemental Material

sj-docx-1-ine-10.1177_15910199221140962 - Supplemental material for Deep learning prediction of stroke thrombus red blood cell content from multiparametric MRI Supplemental material, sj-docx-1-ine-10.1177_15910199221140962 for Deep learning prediction of stroke thrombus red blood cell content from multiparametric MRI by Spencer D Christiansen, Junmin Liu, Maria Bres Bullrich, Manas Sharma, Melfort Boulton, Sachin K Pandey, Luciano A Sposato and Maria Drangova in Interventional Neuroradiology
