# Supplementary material for: Deep learning prediction of stroke thrombus red blood cell content from multiparametric MRI
Source: Interv Neuroradiol. 2022 Nov 28;30(4):541–9. doi: 10.1177/15910199221140962 (PMC11483724; doi:10.1177/15910199221140962)
Supplement: sj-docx-1-ine-10.1177_15910199221140962 - Supplemental material for Deep learning prediction of stroke thrombus red blood cell content from multiparametric MRI [file sj-docx-1-ine-10.1177_15910199221140962.docx]

**SUPPLEMENTARY MATERIAL**


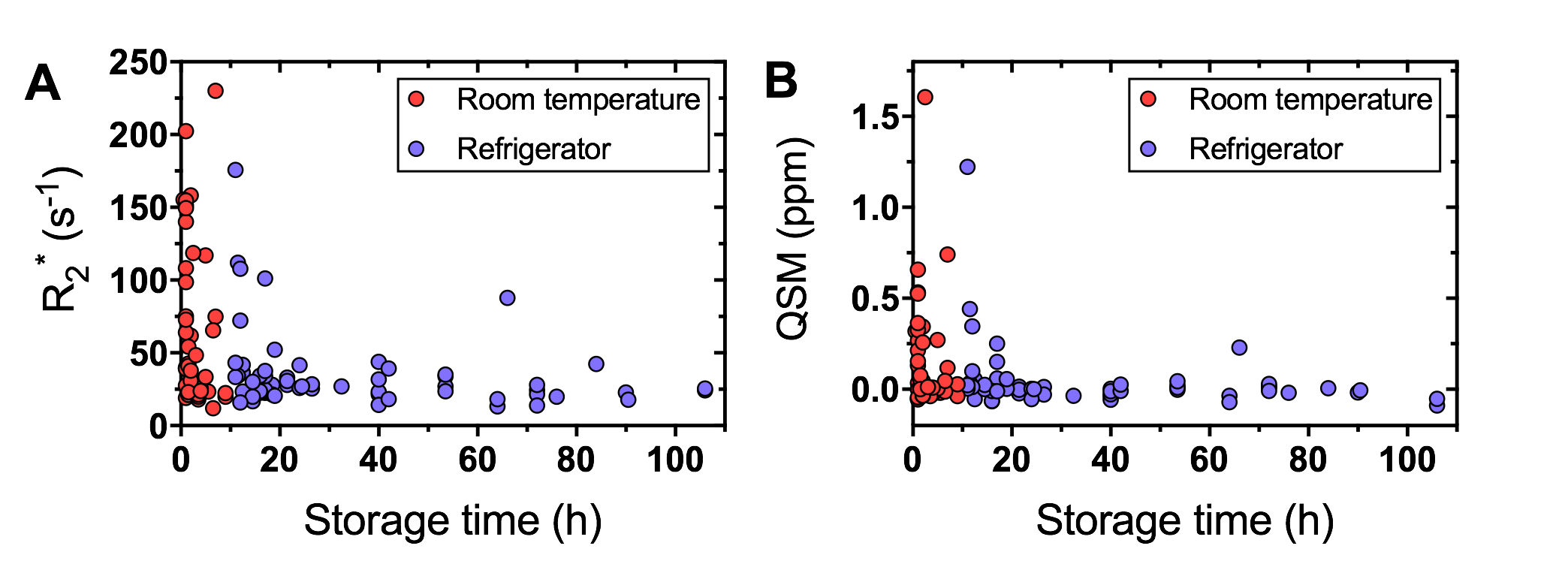


**Figure 1.** Effect of storage method and time between thrombus retrieval and MR imaging on mean thrombus (A) R_2_^*^ and (B) QSM values. Thrombi were either stored at room temperature and scanned within the same workday, or stored in a fridge until the next available workday. Two-tailed T-tests between the two groups R_2_^*^ and QSM values yielded significant differences with *P* < 0.001 and *P* = 0.02, respectively.

**
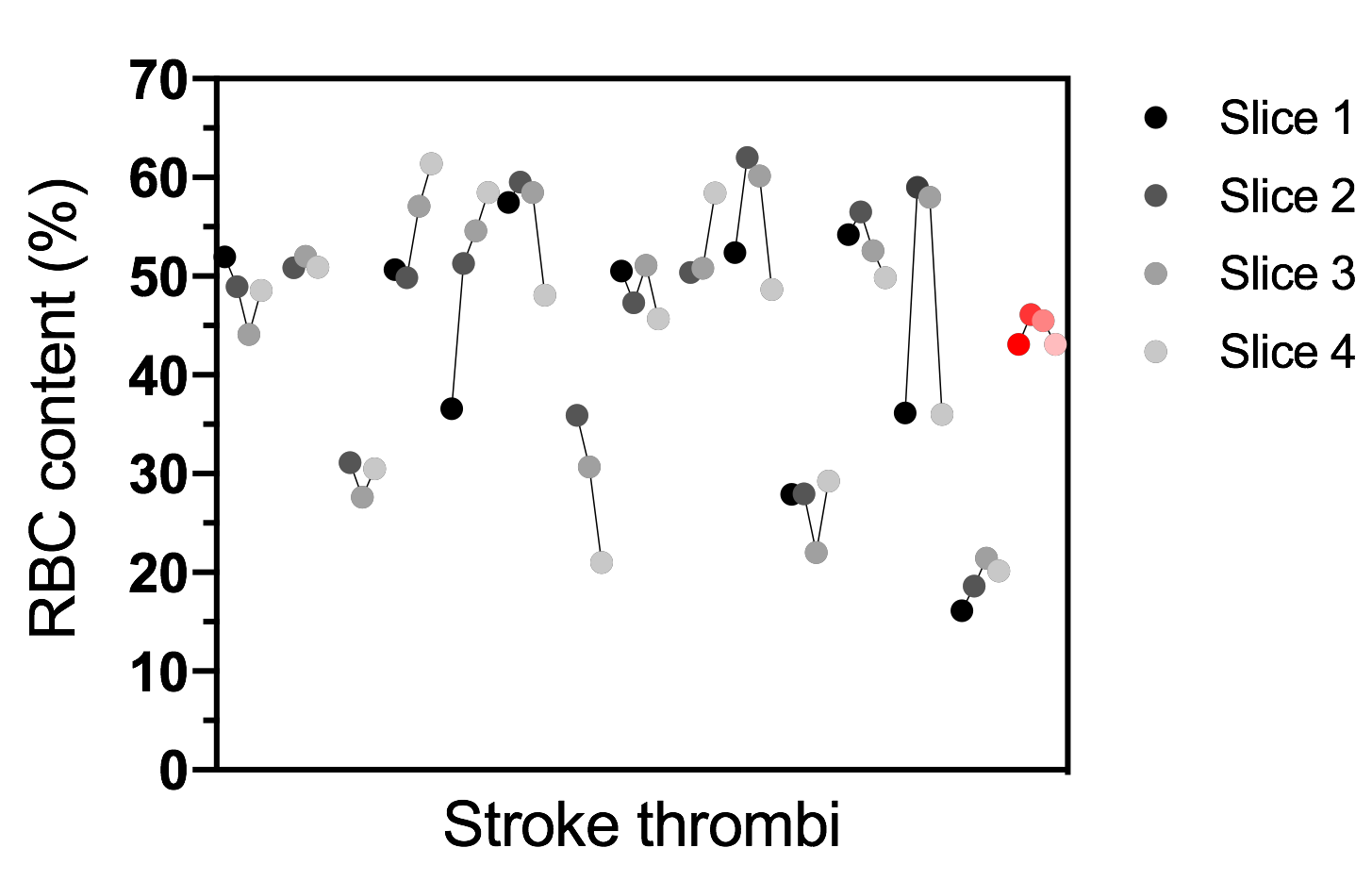
**

**Figure 2.** Thrombus multi-section histological results. Mean RBC content is shown at 3 or 4 evenly spaced intervals through the retrieved stroke thrombi. Red dots indicate the overall average RBC content of all thrombi at each slice, and demonstrate no intra-thrombus trend.

**
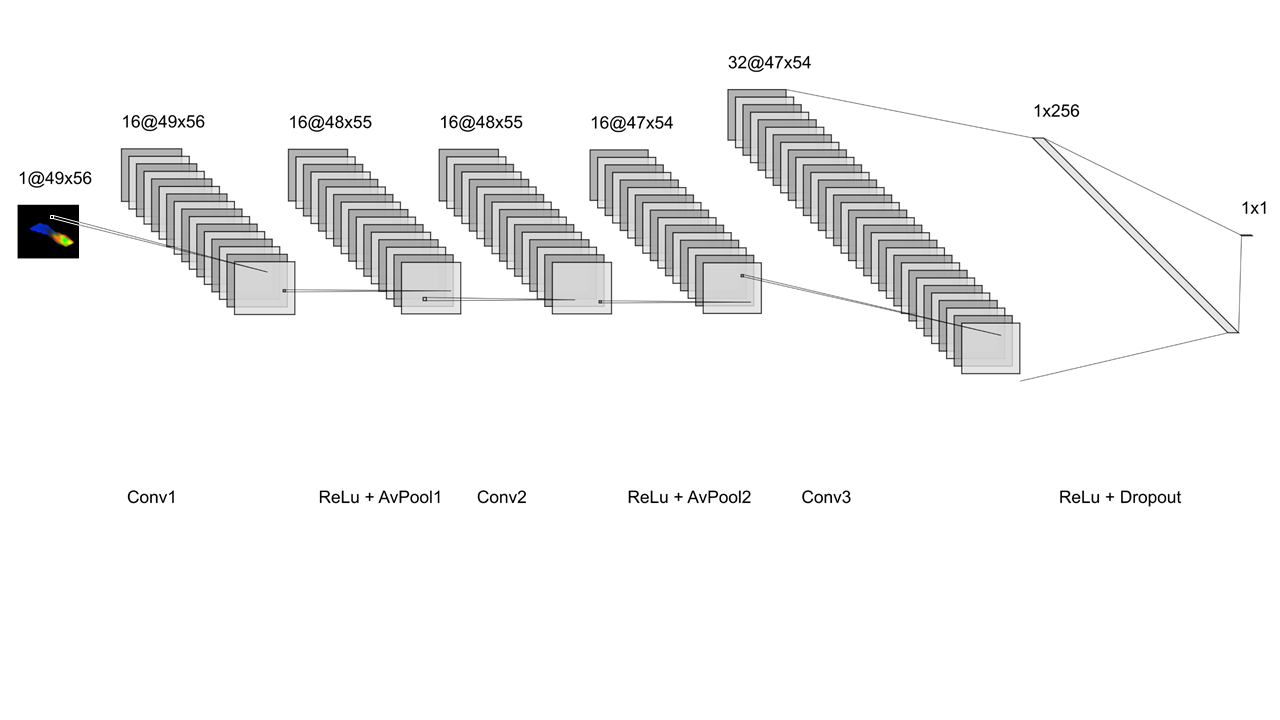
**

**Figure 3.** Schematic diagram of the CNN. Three-dimensional RGB images consisting of segmented, normalized thrombus R_2_^*^, QSM and GRE magnitude images are fed into a network consisting of 3 convolutions layers (3×3, stride of 1) with average pooling layers (2×2, stride of 1) in between and a dropout layer at the end, before a final fully-connected layer with a single output connected to a regression layer. Zero-padding is employed to retain image size in the convolutional layers.

**Table 1.** Optimal network training parameters determined through a grid search optimized over mean absolute error.

| Training set | Batch size | Learning rate | Epochs | λ | Dropout |
| --- | --- | --- | --- | --- | --- |
| Original | 8 | 0.001 | 8 | 0.01 | 0 |
| Augmented | 12 | 0.001 | 4 | 0.01 | 0.2 |

λ: L2 regularization parameter.


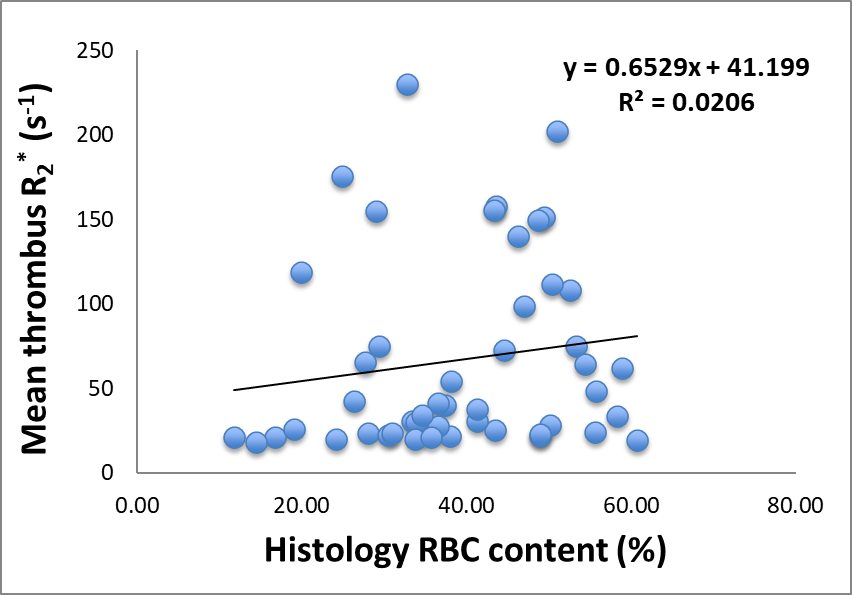

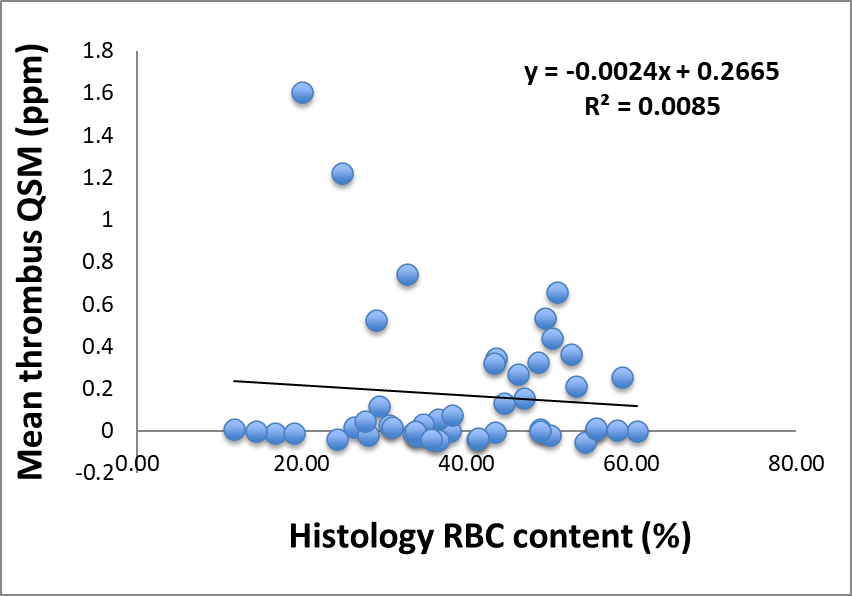


**Figure 4.** Correlation between mean thrombus R_2_^*^ (left) and QSM (right) imaging values and RBC content.


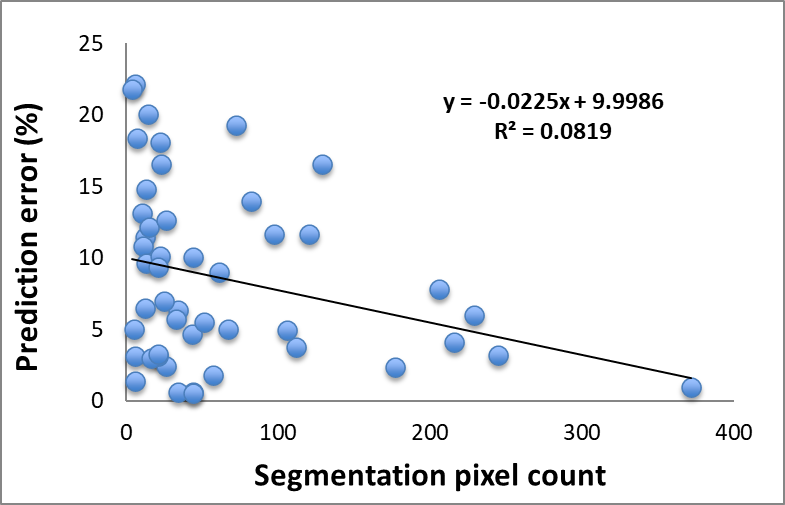

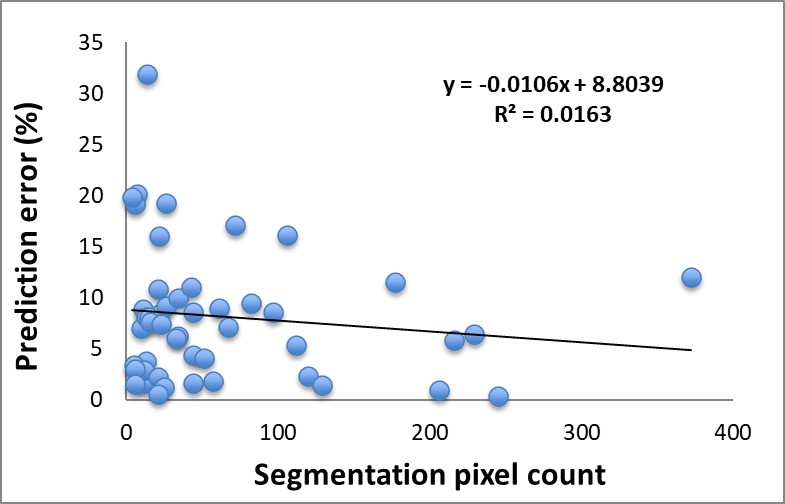


**Figure 5.** Correlation between mean thrombus size and absolute RBC content prediction error in the networks trained on the original (left) and augmented (right) datasets.

**Table 2.** Median thrombus imaging summary values for all subsets of examined thrombi. RBC-rich and poor thrombi were defined as having RBC content higher or lower than the median thrombus RBC content value (38%), respectively.

| Subset | R_2_^*^ median (IQR) [s^-1^] | QSM median (IQR) [ppm] |
| --- | --- | --- |
| All thrombi | 39 (24 – 101) | 0.017 (-0.007 – 0.26) |
| RBC-poor | 29 (22 – 48) | 0.012 (-0.012 – 0.05) |
| RBC: 20 – 45% | 38 (25 – 74) | 0.027 (-0.016 – 0.12) |
| RBC-rich | 63 (30 – 119) | 0.10 (0.001 – 0.32) |

IQR: interquartile ratio; QSM: quantitative susceptibility mapping; RBC: red blood cell.
